# Hydroxyethyl starch versus other fluids for non-septic patients in the intensive care unit: a meta-analysis of randomized controlled trials

**DOI:** 10.1186/s13054-015-0833-9

**Published:** 2015-03-19

**Authors:** Bin He, Bo Xu, Xiaoxing Xu, Lixia Li, Rongrong Ren, Zhiyu Chen, Jian Xiao, Yingwei Wang, Bin Xu

**Affiliations:** Department of Anesthesiology and SICU, Xinhua Hospital, Shanghai Jiaotong University, School of Medicine, Kongjiang Road 1665, Shanghai, 200092 China; Department of Epidemiology, Shanghai Jiaotong University, School of Medicine, Kongjiang Road 1665, Shanghai, 200092 China; Pharmaceutical Department, Xinhua Hospital, Shanghai Jiaotong University, School of Medicine, Kongjiang Road 1665, Shanghai, 200092 China; Department of Hepato-Biliary-Pancreatic Surgery, Second Military Medical University, Fengyang Road 415, Shanghai, 200003 China; Department of Hepato-Biliary-Pancreatic Surgery, Shanghai Tenth People’s Hospital, Tongji University, School of Medicine, Yanchang Road 301, Shanghai, 200072 China

## Abstract

**Introduction:**

Use of hydroxyethyl starch (HES) in septic patients is reported to increase the mortality and incidence of renal replacement therapy (RRT). However, whether or not use of HES would induce the same result in non-septic patients in the intensive care unit (ICU) remains unclear. The objective of this meta-analysis was to evaluate 6% HES versus other fluids for non-septic ICU patients.

**Methods:**

Randomized controlled trials (RCTs) were searched from Pubmed, OvidSP, Embase database and Cochrane Library, published before November, 2013. A meta-analysis was made on the effect of 6% HES versus other fluids for non-septic ICU patients, including mortality, RRT incidence, bleeding volume, red blood cell (RBC) transfusion and fluid application for non-septic patients in ICU.

**Results:**

Twenty-two RCTs were included, involving 6,064 non-septic ICU patients. Compared with the other fluids, 6% HES was not associated with decreased overall mortality (RR = 1.03, 95%CI: 0.09 to 1.17; *P* = 0.67; *I*^*2*^ = 0). There was no significant difference in RRT incidence, bleeding volume and red blood cell transfusion between 6% HES group and the other fluid groups. However, patients in HES group received less total intravenous fluids than those receiving crystalloids during the first day in ICU (SMD = −0.84; 95%CI: −1.39 to −0.30; *P* = 0.003, *I*^*2*^ = 74%).

**Conclusions:**

This meta-analysis found no increased mortality, RRT incidence, bleeding volumes or RBC transfusion in non-septic ICU patients, but the sample sizes were small and the studies generally were of poor quality.

**Electronic supplementary material:**

The online version of this article (doi:10.1186/s13054-015-0833-9) contains supplementary material, which is available to authorized users.

## Introduction

Since the 1970s, hydroxyethyl starch (HES) has been widely used for volume expansion therapy. A cross-sectional study reported that about 37.1% patients in the ICU received daily plasma volume expansion, mostly using HES [1]. Another study showed that rapid intravenous infusion of HES increases cardiac output and expands blood volume more effectively than crystalloids [2]. Numerous reviews without meta-analyses also support the clinical application of HES [3,4].

However, the safety of HES for plasma volume expansion is still under research, especially as many studies by Boldt have been retracted due to scientific misconduct. Recently, three large-sample randomized controlled trials (RCTs) [5-7] and meta-analyses [8-10] have indicated that HES increases the need for renal replacement therapy (RRT) in critically ill patients and mortality in sepsis patients. The statement issued by the European Society of Intensive Care Medicine (ESICM) recommends that products containing HES should not be used in septic patients (1B), other intensive care patients at a high risk of acute kidney injury (AKI) (1C) or patients with head injury or intracranial bleeding (1C) [11]. However, another international multi-center large-sample RCT reported the use of colloids (including HES, gelatin, dextran and albumin) versus any crystalloids for volume expansion therapy to decrease 90-day mortality without increasing the need for in ICU patients [12]. Hence, we wanted to further study the effect of the use of HES in ICU patients.

There are also many non-septic patients in the ICU who require volume expansion therapy, such as those with acute hypovolemia arising from trauma, bleeding or surgery. So far, there is a lack of effective and comprehensive evidence-based trials focusing on the safety of HES for non-septic patients in the ICU. However, Zarychanski *et al*. [8] have reported on the use of HES in critically ill patients, and performed subgroup analyses to compare trials of septic versus non-septic patients in their meta-analysis, the results were inconsistent between subgroups. After exclusion of the Boldt’ papers HES was shown to significantly increase the risk of mortality in all critically ill patients. However, HES did not increase the mortality in the non-septic subgroup, which was contradictory to the result obtained from the septic subgroup in their paper. Furthermore, subgroup analysis was performed only for mortality; AKI and RRT incidence were not reported in the non-septic subgroup. Mutter *et al*. [9] made a systematic review to assess the effects of HES versus other fluids on kidney function in all patient populations. HES products were found to increase the risk in AKI and RRT in all patients. Surprisingly, Mutter *et al*. detected a significant decrease in risk and injury of renal function according to the risk, injury, failure, loss of kidney function and end-stage kidney disease (RIFLE) criteria in the non-septic subgroup treated with HES versus other fluids [9].

Two meta-analyses published recently demonstrated that use of HES for volume expansion therapy during surgery was not associated with increased postoperative mortality or RRT use [13,14], which differed from the studies focusing on septic patients. However, Cittanova *et al*. reported that HES significantly increases serum creatinine concentrations during the first 8 days after transplantation in kidney-transplant recipients [15]. These results suggest that HES could have different effects on different diseases. Therefore, whether or not use of HES in non-septic ICU patients could induce a result similar to that seen in septic ICU patients needs to be further confirmed. The aim of the present study was to evaluate the impact of 6% HES on mortality, RRT use, bleeding volume, red blood cell (RBC) transfusion and fluid application among non-septic patients in the ICU.

## Materials and methods

According to the methodology recommended by the Cochrane Collaboration [16], we included related RCTs for analyzing the safety of 6% HES for non-septic patients in the ICU. The primary endpoints were overall mortality and use of RRT, and the secondary endpoints were bleeding volume, RBC transfusion and fluid application. We reported the meta-analysis according to the preferred reporting items for systematic reviews and meta-analyses (PRISMA) criteria [17].

Studies were selected if they met the following criteria: 1) RCTs; 2) patient age ≥18 years; 3) studies consisting of a group of patients in whom 6% HES was used, and a control group receiving other intravenous fluids in the ICU; and 4) subgroups of non-septic patients who were reported to have received 6% HES and other intravenous fluids in the ICU. Studies were excluded if they had any of the following characteristics: 1) septic patients as research subjects; 2) no group receiving 6% HES; 3) no data available and 4) Boldt’s research studies.

### Search strategy

We searched Pubmed, OvidSP, Embase database and the Cochrane Library, including reference lists of relevant clinical trials, systematic reviews and meta-analyses published before November 2013, and that met the above criteria. The term in MeSH was “Hetastarch” and related free words were also searched such as “hydroxyethylstarch”, “HES”, “Tetraspan”, “Voluven” and so on. The search was limited by “RCTs”, “human” and “adult”. “Language” was not a restricted searching condition. Details are provided in Additional file 1.

### Data extraction

Two reviewers (BH, BX) independently screened the results of the retrieved and acquired full texts that met the above criteria. For each acquired article, the two reviewers independently extracted the valid data, including overall mortality, RRT use, bleeding volume, RBC transfusion, and fluid application during the first day in ICU. A third reviewer (XX) would arbitrate in the event of any disagreement between the two reviewers.

### Risk of bias assessment and study quality

The Cochrane Collaboration risk-of-bias tool [16] was used to evaluate the internal validity of the included articles. The tool contained the following items: generation of random sequence, allocation concealment, blinding, incomplete data reporting, selective reporting results and other problems that could put the study at a risk of bias. Quality assessment was evaluated using mthe odified Jadad score [18]. The scale is a score from 0 to 7 (highest level of quality) according to generation of random sequence, allocation concealment, blinding, and withdrawals of clinical trials. High quality was defined by a Jadad score of 4 to 7; low quality was defined by a Jadad score ≤3.

### Statistical analysis

Review Manager (RevMan, version 5.2) was used to analyze the included studies and data. Standard mean difference (SMD) was used for pooling continuous data. When median and extreme values were presented in the original articles, these data were converted into mean and SD according to relevant formulas [19]. If median and quartile range were reported, mean value and SD were estimated by the method provided in the 7.7.3.5 section of the Cochrane Handbook [16]. If the HES groups or control groups included more than one group, the respective data were pooled (weighted estimate) according to the Cochrane Handbook (7.7.3.8 section) [16]. For non-continuous data, relative risk (RR) was adopted. Heterogeneity was quantified using the *I*^2^-test [20]. The fixed effects model was selected if there was no heterogeneity (*I*^2^ < 50%), and the random effects model was selected in the event of 50% ≤ *I*^2^ < 75%. A sensitivity analysis or subgroup analysis was performed to exclude the heterogeneity if *I*^2^ was ≥75%, otherwise meta-analysis was not carried out. Publication bias was tested using funnel plots and the Egger’s test [21]. Two-sided tests were performed with a significant difference at *P* <0.05.

## Results

### RCTs included

**Figure 1:**
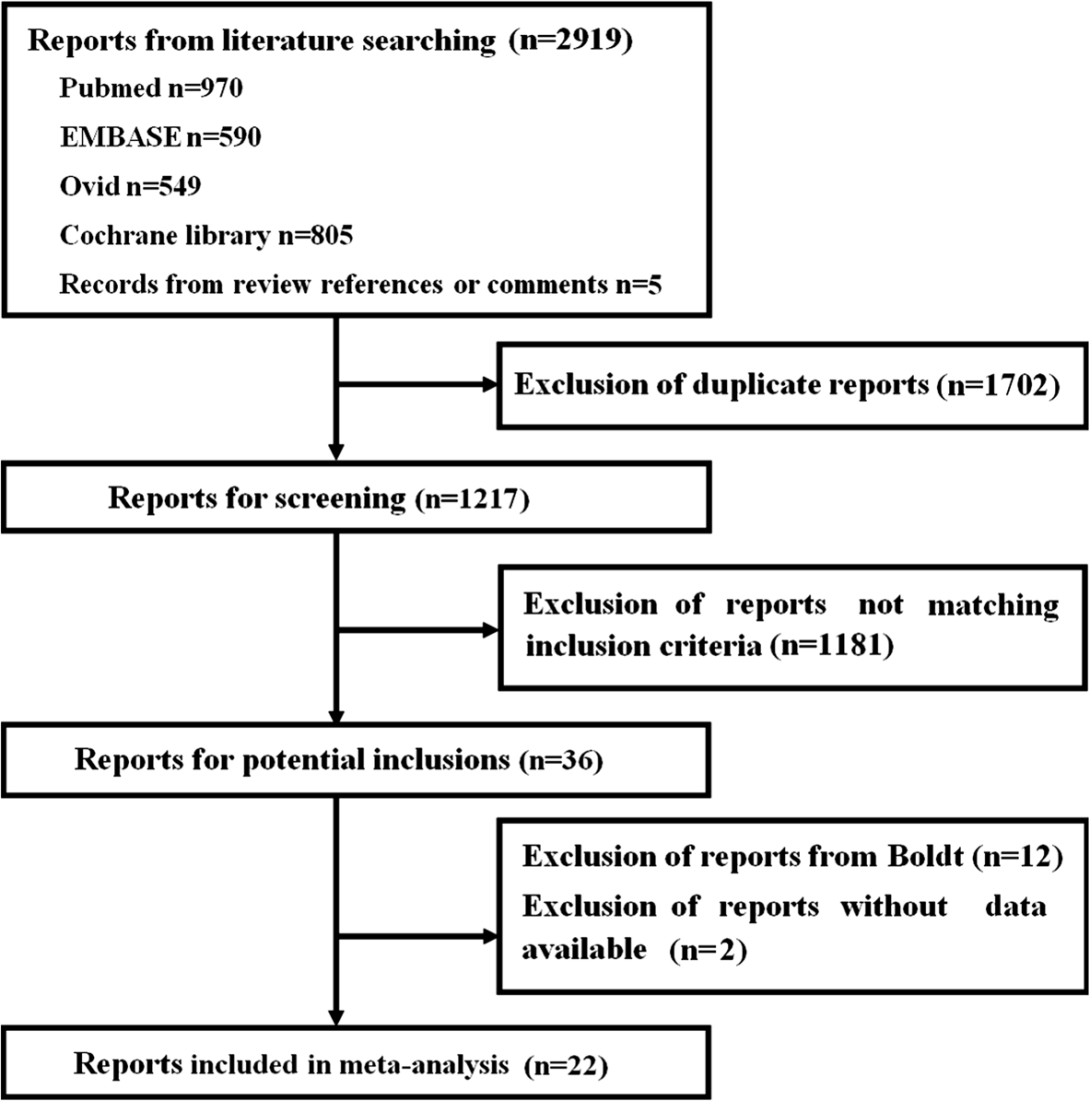
**Flow diagram of this meta-analysis.**

The flow diagram of this meta-analysis is presented in Figure 1. A total of 2,919 articles were retrieved, of which 2,897 were excluded. Finally, 22 eligible RCTs were included in this meta-analysis according to the inclusion and exclusion criteria.

### Characteristics of the included studies and study quality

The characteristics of the 22 included RCTs are shown in Table 1. Of these, 14 RCTs reported patients who had undergone complex surgical procedures needing volume expansion therapy in the ICU [22-35], and 8 RCTs reported patients with other diseases (such as cardiac arrest, trauma, and so on) who received volume expansion therapy in the ICU [6,36-42]. Two RCTs reported data for a non-septic subgroup [6,38], and we included these data. These studies used different indicators to evaluate whether patients reached circulation stabilization. For example, one study used cardiac index [22], some chose urine and central venous pressure [37,41,42], and others combined several indicators [23,24,28-32,39].

**Table 1:** **Characteristics of articles included, patient diagnosis, number of participants, interventions, and related details of reports**

**Author, year**	**Diagnosis**	**Patients (n)**	**Intervention**		**Control**		**Total dose of hydroxyethyl starch** **(HES)**	**Volume expansion goals**	**Intervention period**	**PD or CA**
			**Study fluids**	**n1**	**Control fluids n2**					
Ley, 1990 [22]	After cardiac surgery	21	6% HES 450/0.7 11		Normal saline	10	Up to 1.5 L	CI >2.0 L/min/m^2^	NS	PD
Beards,1994 [36]	Hypovolemia	25	6% HES 450/0.7 13		Gelatin	12	500 mL bolus	NS	15 to 30 minutes	PD
Van der Linden, 2005 [23]	After cardiac surgery	132	6% HES 130/0.4	64	Gelatin	68	Maximum 50 mL/kg/day	PAOP, 8 to 15 mmHg	NS	PD
								CI >2.5 L/min/m^2^		
								Urine >0.5 mL/kg/h		
Chen, 2006 [37]	Burn	66	6% HES 130/0.4	33	Plasma	33	2328 ± 271 mL	CVP 7 to 14 mmHg	48 h	CA
								Urine >0.5 mL/kg/h		
Mahmood, 2007 [24]	After aortic aneurysm surgery	62	6% HES 130/0.4	21	Gelatin	20	Surgery, 3 ml/kg/h	MAP >85 mmHg	Surgery and ICU	PD
			6% HES 200/0.62	21			CU, as needed	CVP 8 to 10 mmHg		
Godet, 2008 [25]	After abdominal aortic surgery	65	6% HES 130/0.4	32	Gelatin	33	As needed	Doctor’s judgement	Surgery and 6 d in ICU	PD
Mukhtar, 2009 [26]	After transplantation surgery	40	6% HES 130/0.4	20	Albumin	20	Maximum 50 mL/kg/day	CVP, 5 to 7 mmHg	Surgery and 4 d in ICU	PD
Ooi, 2009 [27]	After cardiac surgery	90	6% HES 130/0.4	45	Gelatin	45	As needed	CVP, 10 to 14 mmHg	NS	PD
Schramko, 2009 [28]	After cardiac surgery	45	6% HES 130/0.4	15	Albumin	15	NS	PAWP, 10 to 14 mmHg	NS	PD
			6% HES 200/0.62	15				CI >2.0 L/min/m^2^		
Choi, 2010 [29]	After abdominal aortic surgery	36	6% HES 130/0.4	18	Albumin	18	Maximum 20 mL/kg in ICU	PAOP, 10 to 14 mmHg	NS	PD
								CI >2.0 L/min/m^2^		
								Urine >0.5 mL/kg/h		
Gondos, 2010 [38]	Hypovolemia	106	6% HES 130/0.4	26	RL	28	10 mL/kg	NS	NS	PD
					Gelatin	25				
					Albumin	27				
Heradstveit, 2010 [39]	After cardiac arrest	19	6% HES 200/0.5	10	RL	9	maximum 500 mL/24 h	MAP >60 mmHg	Observation period	PD
								HR, 60 to 100/min		
								CVP, 8 to 12 mmHg		
Inal, 2010 [40]	Hypovolemia	30	6% HES 130/0.4	15	Gelatin	15	500 mL	NS	NS	PD
Schramko, 2010 [30]	After cardiac surgery	45	6% HES 130/0.4	15	RL	15	28 mL/kg	PCWP, 10 to15 mmHg	16 to 20 h in ICU	PD
					Gelatin	15		CI > 2.0 L/min/m^2^		
Du, 2011 [41]	Acute pancreatitis	41	6% HES 130/0.4	20	RL	21	as needed	CVP, 8 to 15 mmHg	8 d in hospital	CA
							1:3 with saline	Urine >0.5 mL/kg/h		
								SBP >90 mmHg		
James, 2011 [42]	Trauma	109	6% HES 130/0.4	56	Normal saline	53	500 mL boluses	CVP >12 mmHg	15 minutes	PD
								Urine >0.5 mL/kg/h		
Lee, 2011 [31]	After cardiac surgery	106	6% HES 130/0.4	53	Crystalloid	53	Maximum 50 mL/kg/d	CI >2.2 L/min/m^2^	NS	PD
								SvO2 > 60%		
								Urine >0.5 mL/kg/h		
Yang, 2011 [32]	After hepatectomy	81	6% HES 130/0.4	26	RL	25	1,000 mL/d*3d	CVP, 5 to 9 mmHg	5 d	CA
					Albumin	30	500 mL/d*2d	MAP, 60 to 80 mmHg		
Myburgh, 2012 [6]	Non-septic patients	4720	6% HES 130/0.4	2337	Normal saline	2383	500 mL bolus, maximum 50 mL/kg/d	NS	90 d	CA
Alavi, 2012 [33]	After cardiac surgery	92	6% HES 130/0.4	32	RL	29	As needed	CVP, 7 to 14 mmHg	Surgery and ICU	PD
					Gelatin	31				
Nagpal D, 2012 [34]	After cardiac surgery	70	6% HES 130/0.4	35	Crystalloid	35	1 to 3.0 L/d	NS	NS	PD
Kimenai, 2013 [35]	After cardiac surgery	60	6% HES 130/0.4	30	Gelatin	30	NS	NS	NS	PD

RL, Ringer lactate; n, number of overall patients; n1, number of patients in intervention group; n2 number of patients in control group; NS, not stated; CI, cardiac index; PAOP, pulmonary artery occlusive pressure; CVP, central venous pressure; MAP, mean arterial pressure; PAWP, pulmonary artery wedge pressure; HR, heart rate; PCWP, pulmonary capillary wedge pressure; SBP systolic blood pressure; SvO2, mixed venous oxygen saturation; PD, published data; CA, connected with author successfully.

Among our included articles, 14 RCTs [6,23-27,32,34,36,38-42] reported the overall mortality; 9 RCTs [24-27,31,34,37,41,42] reported the incidence of RRT; 10 RCTs [22-24,27-31,33,35] reported bleeding volume after surgery; 10 RCTs [22,24-28,30,31,33,35] reported RBC transfusion after surgery; and 9 RCTs [23,27-30,32,33,37,42] reported fluid application during the first day in ICU (Additional file 2: Table S1). According to the modified Jadad score, high quality was determined in 13 studies, and low quality was determined in 9 studies (Table 2).

**Table 2:** **Risk of bias and literature quality**

**Trials**	**Random sequence generation**	**Allocation concealment**	**Blinding**	**Incomplete outcome data treatment**	**Selective outcome reporting**	**Other bias**	**Jadad score**
High Quality							
Van der Linden, 2005 [23]	Low	Unclear	High	Low	Low	Low	4
Mahmood, 2007 [24]	Low	Low	High	Low	Low	Low	5
Godet, 2008 [25]	Low	Low	High	Low	Low	High	4
Mukhtar, 2009 [26]	Unclear	Low	Unclear	Low	Low	Unclear	4
Schramko, 2009 [28]	Unclear	Low	Unclear	Low	Low	Low	4
Gondos, 2010 [38]	Unclear	Low	Unclear	Low	Low	Low	4
Schramko, 2010 [30]	Unclear	Low	High	Low	Low	Low	4
Du, 2011 [41]	Low	Unclear	Unclear	High	High	Low	4
James, 2011 [42]	Low	Low	Low	High	Low	Low	6
Myburgh, 2012 [6]	Low	Low	Low	Low	Low	Low	7
Alavi, 2012 [33]	Unclear	Low	Low	Low	Low	Low	4
Nagpal, 2012 [34]	Low	Low	Low	Unclear	Low	Low	5
Kimenai, 2013 [35]	Low	Unclear	High	Low	Low	Low	4
Low Quality							
Ley, 1990 [22]	Unclear	Unclear	Unclear	Low	Low	Unclear	2
Berard, 1994 [36]	Unclear	Unclear	Unclear	Low	Low	Low	2
Chen, 2006 [37]	Low	Unclear	High	Low	Low	Unclear	3
Ooi, 2009 [27]	Unclear	Unclear	High	Low	Unclear	Low	2
Choi, 2010 [29]	Low	Unclear	Unclear	Low	Low	Low	3
Heradstveit, 2010 [39]	Unclear	Unclear	High	Low	Low	High	2
Inal, 2010 [40]	Unclear	Unclear	Unclear	Low	Unclear	Low	2
Lee, 2011 [31]	Unclear	Unclear	High	Low	Low	Low	2
Yang, 2011 [32]	Low	Unclear	Unclear	High	Low	Low	3

### Overall mortality

**Figure 2:**
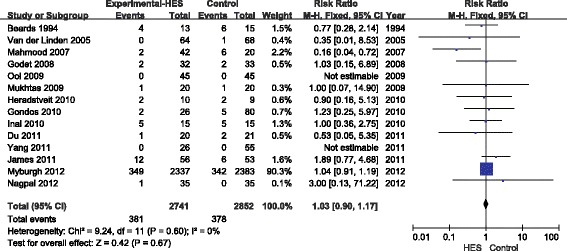
**Forest plot of pooled risk ratio for overall mortality.** HES, hydroxethyl starch. M-H, Mantel-Haenszel.

**Figure 3:**
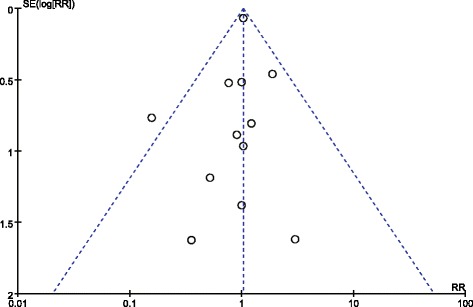
**Funnel plot of overall mortality.** RR, relative risk. SE, standard error.

A total of 14 articles reported the overall mortality, involving 5,593 patients. Compared with the other types of fluids (crystalloids, gelatine or albumin), the use of 6% HES was not associated with decreased overall mortality (RR = 1.03, 95% CI 0.90 to 1.17; *P* = 0.67; *I*^2^ = 0) (Figure 2). Publication bias was not found by the Egger’s test (*P* = 0.85) or funnel plots (Figure 3).

### Renal replacement therapy

**Figure 4:**
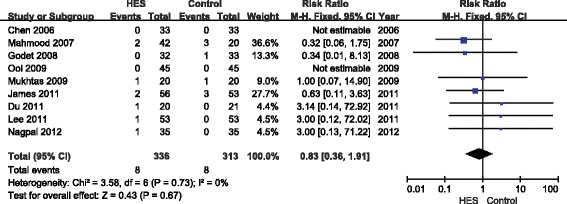
**Forest plot of pooled risk ratio for use of renal replacement therapy.** HES, hydroxethyl starch. M-H, Mantel-Haenszel.

**Figure 5:**
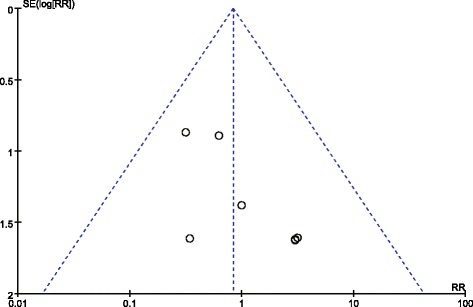
**Funnel plot of incidence for renal replacement therapy.** RR, relative risk. SE, standard error.

Nine articles reported the incidence of RRT, showing that 6% HES did not increase RRT use as compared with the other fluids (RR = 0.83; 95% CI 0.36 to 1.91; *P* = 0.67; *I*^2^ = 0%) (Figure 4). Funnel plots showed no publication bias (Figure 5), and the *P*-value from Egger’s test was 0.58.

### Bleeding volume and RBC transfusion

**Figure 6:**
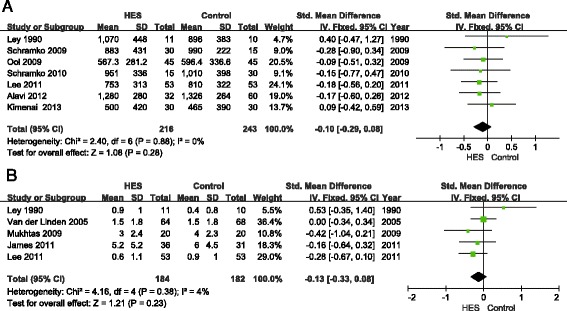
**Forest plots of pooled estimates for bleeding volume and red blood cell transfusion. (A)** Bleeding volume after surgery. **(B)** Red blood cell transfusion (unit). HES, hydroxethyl starch. IV, Inverse Variance.

Bleeding volume was reported in 10 articles. Of these, we only pooled seven articles [22,27,28,30,31,33,35] in this meta-analysis, which all reported bleeding volume after surgery. Three articles [23,24,29] were excluded, as bleeding volume was reported from surgery or during several periods after surgery. Data from Schramko *et al*., Alavi *et al*. and Kimenai *et al*. were converted into mean and SD according to the method above [16,19]. There was no significant difference in bleeding volume between the 6% HES group and other fluid groups (SMD = −0.10, 95% CI −0.29 to 0.08; *P* = 0.28; *I*^2^ = 0%) (Figure 6A). Egger’s test showed no publication bias (*P* = 0.35).

Nine articles reported RBC transfusion, and five articles [22,23,26,31,42] reporting RBC transfusion after surgery were included and showed no significant difference between the HES group and other fluids group (SMD = −0.13; 95% CI −0.33 to 0.08; *P* = 0.23; *I*^2^ = 4%) (Figure 6B). Egger’s test showed no publication bias (*P* = 0.51). Four articles were excluded because two of them [28,33] only reported overall volume of RBC transfusion, and the other two [24,26] reported RBC transfusion before surgery. Data from three articles were transformed [23,26,42].

### Fluid application

**Figure 7:**
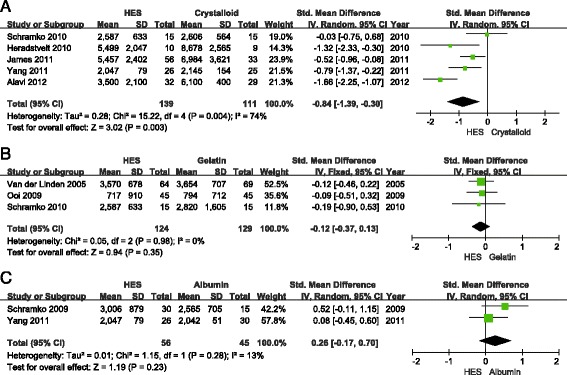
**Forest plots of pooled estimates for fluid application (mL) during the first day in the ICU. (A)** Hydroxethyl starch (HES) versus crystalloid. **(B)** HES versus gelatin. **(C)** HES versus albumin. IV, Inverse Variance.

Fluid application during the first day in ICU was reported in nine articles, but there was significant data heterogeneity (*I*^2^ = 94%). Knowing that colloids and crystalloids have different effects on volume expansion, a subgroup analysis was performed by the type of fluid used. Patients receiving 6% HES needed fewer total intravenous fluids than those receiving crystalloids (SMD = −0.84, 95% CI −1.39 to −0.30; *P* = 0.003; *I*^2^ = 74%) (Figure 7A). There was no significant difference between the 6% HES group and albumin group (SMD = 0.26, 95% CI −0.17 to 0.70; *P* = 0.23; *I*^2^ = 13%) (Figure 7C). In this subgroup, data from Choi *et al*. [29] were excluded, because they were reported in several periods during the first day in ICU, and we failed to obtain the data from the authors. Although subgroup analysis was carried out, there remained great heterogeneity in the fluid application between the HES group and the gelatin group. A sensitivity analysis identified that the trial from Alavi *et al*. [33] was responsible for the heterogeneity. When their data were excluded, there was no significant difference in fluid application between the HES group and the gelatin group (SMD = −0.12, 95% CI −0.37 to 0.13; *P* = 0.35; *I*^2^ = 0%) (Figure 7B).

### Length of ICU and hospital stay

Eleven articles reported the length of ICU stay, and nine articles reported the length of hospital stay. However, significant heterogeneity was detected in both (ICU stay: *I*^2^ = 85%; hospital stay: *I*^2^ = 78%). As the heterogeneity could not be excluded through subgroup analysis or sensitivity analysis, a description was provided instead of performing a meta-analysis. These data are not shown.

## Discussion

The main finding of this meta-analysis showed that HES was not associated with decreased overall mortality and RRT use. These findings were similar with subgroup analyses of non-septic patients among critically ill patients or among all patients [8,9]. Our results were somewhat different from a previous meta-analysis of fluid therapy in critically ill patients, especially septic patients [43]. One major cause was that the existing data for these patients treated with HES was poor and scarce, which meant the results needed to be verified with more data of high quality. Another reason might also be that the pathophysiological changes in these non-septic patients are different from those in septic patients, and sepsis itself could significantly increase the risk of AKI [44]. Micro leakage of capillary blood vessels was a key pathophysiologic mechanism in sepsis [45], and endothelial glycocalix plays an important role in micro leakage of capillary blood vessels [46]. Steppan *et al*. found that significantly more flaking of the endothelial glycocalix occurred in patients with sepsis than in patients who underwent major abdominal surgery [47]. In addition, sepsis could activate the renin-angiotensin-aldosterone system (RAAS) in renal sympathetic and angiotensin activities, which leads to vasoconstriction in patients with sodium and water retention. As a result, septic patients are predisposed to acute renal failure [44]. Hence, the side effects of HES might be more serious for septic patients than non-septic patients in the ICU.

Although several studies tried to analyze the effect of HES in a non-septic subgroup, the results of their subgroup analyses were always different from their main results [8,9]. In addition, their analyses were always part of the side effects of HES. Hence, we have made a meta-analysis to study the effect HES versus other fluids for non-septic ICU patients, including mortality, RRT use, bleeding volume, RBC transfusion and fluid application. This analysis was relatively comprehensive and with less heterogenicity in non-septic patients. Furthermore, it reminded us to pay more attention to volume expansion therapy in ICU non-septic patients.

The pooled analysis of overall mortality did not display more harm with HES, which was not entirely the same as previous trials [5-7] and meta-analyses [8,10,48,49]. Knowing that the pooled analysis of mortality may be influenced by study quality and the follow-up period, it is difficult to explain this result. On the one hand, HES may have different effects in different diseases [5-7,13,14]. On the other hand, study quality and the follow-up period may induce bias in our meta-analysis, as most included RCTs were small-sample studies and with short follow-up periods. Hence, we should be cautious when selecting fluids for ICU non-septic patients. In addition, the article from CHEST [6] alone accounted for 88.9% of the weighting, and the results, which played an important role in our meta-analysis, might only be suitable for the specific clinical conditions in that study (for example, a small dose of HES was chosen). We have performed a sensitivity analysis, and its exclusion did not influence the significance of the effect on overall mortality (RR = 0.83, 95% CI 0.56 to 1.24; *P* = 0.37; *I*^2^ = 0). However, other included studies were all with very small-scale samples; more high-quality RCTs focusing on 6% HES in non-septic ICU patients are needed to confirm our results.

Efforts were also made to determine the effect of 6% HES on renal function. However different indicators were used to test renal function, such as blood creatinine [32], glomerular filtration rate [30], the incidence of AKI based on different criteria [24,34,42] and RRT use [24-27,31,34,37,41,42]. The result need further confirmation because of the limitations of our included RCTs.

There was no significant difference in bleeding volume and RBC transfusion between the 6% HES group and the other fluid groups. Patients in the HES group received fewer total intravenous fluids than those receiving crystalloids during the first day in ICU, which might mean that 6% HES had a better volume-expansion effect than crystalloids. However, due to the absence of demonstrable benefit, more large-scale RCTs are needed to confirm these results.

The implementation of our meta-analysis is in accordance with the requirements of the Cochrane Collaboration. These requirements include a literature search without language limitations, strict inclusion and exclusion criteria, selection of articles and collection of data by two independent authors, and bias risk evaluation. Indeed, there are several limitations in our meta-analysis. Despite widespread use of HES for more than three decades, RCTs comparing HES with other fluids for ICU non-septic patients are few, with small sample sizes, and vulnerable to bias. In addition, several control groups in the included studies used gelatin, which clouds or conceals the adverse effects of HES to some extent. Bleeding volume analysis is limited by clinical heterogeneity across studies because patients have undergone different types of surgery.

## Conclusion

Although volume expansion with 6% HES did not seem to increase the mortality or RRT use in non-septic ICU patients, the sample sizes in our meta-analysis were small and the studies generally were of poor quality.

## Key messages

- The safety of HES for non-septic patients in the ICU remains elusive
- Available data from systematic reviews and meta-analyses displayed inconsistent results between septic and non-septic patients using HES
- Use of 6% HES did not seem to increase the mortality or RRT incidence in non-septic ICU patients

## Additional files

Additional file 1:
***A priori*** **design, electronic search strategy and studies excluded from this review.**
Additional file 2: Table S1. Observation period for outcomes.

## Abbreviations

AKI: acute kidney injury
ESICM: European Society of Intensive Care Medicine
HES: hydroxyethyl starch
RAAS: renin-angiotensin-aldosterone system
RBC: red blood cells
RCT: randomized controlled trial
RIFLE criteria: risk injury, failure, loss of kidney function and end-stage kidney disease
RR: relative risk
RRT: renal replacement therapy
SMD: standard mean difference
RBC: red blood cell
