# Supplementary material for: Hydroxyethyl starch versus other fluids for non-septic patients in the intensive care unit: a meta-analysis of randomized controlled trials
Source: Crit Care. 2015 Mar 19;19(1):92. doi: 10.1186/s13054-015-0833-9 (PMC4404666; doi:10.1186/s13054-015-0833-9)
Supplement: Additional file 1: — A priori design, electronic search strategy and studies excluded from this review. [file 13054_2015_833_MOESM1_ESM.doc]

**Additional file 1: A priori design, electronic search strategy and excluded studies from this review**

A: A priori design

Title: Serious adverse events associated with the use of hydroxyethyl starch for volume expansion for non-septic patients in ICU.

Review question(s): Use of hydroxyethyl starch (HES) in septic patients was reported to increase mortality and incidence of renal replacement therapy (RRT). Are these adverse events of HES the same as non-septic patients in ICU?

Searches:

We searched Pubmed, OvidSP, Embase database and Cochrane Library, including reference lists of relevant clinical trials, systematic reviews and meta-analyses published before November, 2013 that met the above criteria. The term in MeSH was "Hetastarch" and related free words were also searched such as "hydroxyethylstarch", "HES", "Tetraspan", "Voluven" and so on. The search was limited by “randomized controlled trials”, “human” and “adult”. “Language” was not a restricted searching condition.

Included criteria: 1) Randomized controlled trials; 2) age≥18 years; 3)Intensive care units patients receiving volume expansion therapy; and 4) one group using 6% HES and control groups receiving other intravenous fluids;

Excluded criteria: 1) septic patients as research subjects; 2) no group receiving 6% HES; 3) Boldt’s research studies.

Data extraction:

Two reviewers independently screened the results of the retrieved and acquired full texts that met the above criteria. For each acquired article, the two reviewers independently extracted the valid data, including mortality, related laboratory test results about renal function, renal replacement therapy incidence, bleeding volume, related laboratory test results about coagulation function, fluid application to maintain functional stabilization of circulation, ICU and hospital stay. A third reviewer would arbitrate in the event of any disagreement between the two reviewers.

Outcomes

Primary outcomes: 1) overall mortality; 2) related laboratory test results about renal function and renal replacement therapy incidence.

Secondary endpoints: 1) bleeding volume and related laboratory test results about coagulation function; 2) fluid application to maintain functional stabilization of circulation; 3) ICU and hospital stay.

Risk of bias assessment and study quality

The Cochrane Collaboration's risk of bias tool was used to evaluate the internal validity of the included articles. The tool contained the following items: generation of random sequence, allocation concealment, blinding, incomplete data reporting, selective reporting results and other problems that could put the study at a risk of bias. Quality assessment was evaluated using modified Jadad Score. The scale is a score from 0 to 7 (highest level of quality) according to generation of random sequence, allocation concealment, blinding, and withdrawals of clinical trials. “High quality” was defined as a Jadad score of 4-7; “low quality” was defined as a Jadad score of <=3.

Statistical analysis

Review Manager (RevMan, version 5.2) was used to analyze the included studies and data. Standard mean difference (SMD) was used for pooling continuous data. When median and extreme values were presented in the original articles, these data were converted into mean and standard deviation according to relevant formulas. If these data were expressed as median and interquartile range, they were translated into mean and standard deviation using method provided by handbook of the Cochrane Collaboration. For non-continuous data, relative risk (RR) was adopted. Heterogeneity was quantified using *I^2^*-test. The fixed effects model was selected if there existed no heterogeneity (*I^2^*<50%), and the random effects model was selected in the event of 50%≤*I^2^*<75%. A sensitivity analysis or subgroup analysis was performed to exclude the heterogeneity if *I^2^* was ≥75%, otherwise meta-analysis was not carried out. Publication bias was tested using funnel plots and the Egger’s test. Two-sided tests were performed with a significant difference at *P*<0.05.

Review team

Dr Bin He, Department of Anesthesiology and SICU, Xinhua Hospital, Shanghai Jiaotong University, School of Medicine.

Dr Bo Xu, Department of Anesthesiology and SICU, Xinhua Hospital, Shanghai Jiaotong University, School of Medicine.

PhD Xiaoxing Xu, Department of Epidemiology, Shanghai Jiaotong University, School of Medicine.

Dr Lixia Li, Pharmaceutical department, Xinhua Hospital, Shanghai Jiaotong University, School of Medicine.

Dr Rongrong Ren, Department of Anesthesiology and SICU, Xinhua Hospital, Shanghai Jiaotong University, School of Medicine.

Dr Zhiyu Chen, Department of Anesthesiology and SICU, Xinhua Hospital, Shanghai Jiaotong University, School of Medicine.

Dr Jian Xiao, Department of Cardiothoracic Surgery, Chang Zheng Hospital, Second Military Medical University.

Dr Bin Xu, Department of Hepato-Biliary-Pancreatic Surgery, Shanghai Tenth People’s Hospital, Tongji University.

Dr Yingwei Wang, Department of Anesthesiology and SICU, Xinhua Hospital, Shanghai Jiaotong University, School of Medicine.

B. The electronic search strategy of this meta-analysis.

a, Pubmed

#1 MeSH descriptor Hetastarch explode all trees

#2 (hydroxyethylstarch or starch or HES or 'hydroxy ethylstarch' or 'hydroxy ethylstarch' or tetrastarch or tetraspan or pentastarch or voluven or hetastarch or HAES-steril or venofundin or Elohes or hextend)

#3 MeSH sepsis explode all trees

#4 (#1 OR #2) not #3

Limit the results with randomized controlled trials, human and adult.

b, Embase

#1. 'hetastarch'/exp OR hetastarch

#2. hydroxyethylstarch or starch or hes or 'hydroxyl ethyl starch' or 'hydroxy ethylstarch' or tetrastarch or tetraspan or pentastarch or voluven or hetastarch or 'haes steril' or venofundin or Elohes or hextend

#3. 'sepsis'/exp

#4. (#1 OR #2) not #3

#5. #4 AND 'randomized controlled trial'/de

#6. #5 AND 'human'/de

#7. #6 AND 'adult'/lim

c, OvidSP

#1. Hetastarch.sh.

#2. (hydroxyethylstarch or starch or HES or hydroxyl ethyl starch or hydroxy ethylstarch or tetrastarch or tetraspan or pentastarch or voluven or hetastarch or HAES-steril or venofundin or Elohes or hextend).tw.

#3. sepsis.sh.

#4. (#1 OR #2) not #3

#5. #4 and "Randomized Controlled Trial" [Publication Type]"

#6. #5 and "Humans" [Subjects]

#7. #6 and "Adult" [Subjects]

d, Cochrane Library

#1. MeSH descriptor: [Hetastarch] explode all trees

#2. hydroxyethylstarch or starch or HES or hydroxyl ethyl starch or hydroxy ethylstarch or tetrastarch or tetraspan or pentastarch or voluven or hetastarch or HAES-steril or venofundin or Elohes or hextend: ti, ab, kw (Word variations have been searched)

#3. #1 OR #2

#4. MeSH descriptor: [Sepsis] explode all trees

#5. #3 not #4

C. Excluded studies from this review.

1. Boldt J, Mayer J, Brosch C, Lehmann A, Mengistu A: Volume replacement with a balanced hydroxyethyl starch (HES) preparation in cardiac surgery patients. *J Cardiothorac Vasc Anesth* 2010, 24:399-407.

2. Boldt J, Ducke M, Kumle B, Papsdorf M, Zurmeyer EL: Influence of different volume replacement strategies on inflammation and endothelial activation in the elderly undergoing major abdominal surgery. *Intensive Care Med* 2004, 30:416-22.

3. Boldt J, Suttner S, Brosch C, Lehmann A, Röhm K, Mengistu A: The influence of a balanced volume replacement concept on inflammation, endothelial activation, and kidney integrity in elderly cardiac surgery patients. *Intensive Care Med* 2009, 35:462-70.

4. Kumle B, Boldt J, Piper S, Schmidt C, Suttner S, Salopek S: The influence of different intravascular volume replacement regimens on renal function in the elderly. *Anesth Analg* 1999, 89:1124-30.

5. Haisch G, Boldt J, Krebs C, Kumle B, Suttner S, Schulz A: The influence of intravascular volume therapy with a new hydroxyethyl starch preparation (6% HES 130/0.4) on coagulation in patients undergoing major abdominal surgery. *Anesth Analg* 2001, 92:565-71.

6. Boldt J, Suttner S, Brosch C, Lehmann A, Röhm K, Mengistu A: Cardiopulmonary bypass priming using a high dose of a balanced hydroxyethyl starch versus an albumin-based priming strategy. *Anesth Analg* 2009,109:1752-62.

7. Boldt J, Brosch C, Ducke M, Papsdorf M, Lehmann A: Influence of volume therapy with a modern hydroxyethylstarch preparation on kidney function in cardiac surgery patients with compromised renal function: a comparison with human albumin. *Crit Care Med* 2007, 35:2740-6.

8. Boldt J, Schöllhorn T, Münchbach J, Pabsdorf M: A total balanced volume replacement strategy using a new balanced hydoxyethyl starch preparation (6% HES 130/0.42) in patients undergoing major abdominal surgery. *Eur J Anaesthesiol* 2007, 24:267-75.

9. Hüttner I, Boldt J, Haisch G, Suttner S, Kumle B, Schulz H: Influence of different colloids on molecular markers of haemostasis and platelet function in patients undergoing major abdominal surgery. *Br J Anaesth* 2000, 85:417-23.

10. Boldt J, Haisch G, Suttner S, Kumle B, Schellhaass A: Effects of a new modified, balanced hydroxyethyl starch preparation (Hextend) on measures of coagulation. *Br J Anaesth* 2002 , 89:722-8.

11. Boldt J, Knothe C, Zickmann B, Andres P, Dapper F, Hempelmann G: Influence of different intravascular volume therapies on platelet function in patients undergoing cardiopulmonary bypass. *Anesth Analg* 1993, 76:1185-90.

12. Boldt J, Brenner T, Lehmann A, Lang J, Kumle B, Werling C: Influence of two different volume replacement regimens on renal function in elderly patients undergoing cardiac surgery: comparison of a new starch preparation with gelatin. *Intensive Care Med* 2003, 29:763-9.

13. Annane D, Siami S, Jaber S, Martin C, Elatrous S, Declère AD, Preiser JC, Outin H, Troché G, Charpentier C, Trouillet JL, Kimmoun A, Forceville X, Darmon M, Lesur O, Reignier J, Abroug F, Berger P, Clec'h C, Cousson J, Thibault L, Chevret S: Effects of fluid resuscitation with colloids vs crystalloids on mortality in critically ill patients presenting with hypovolemic shock: the CRISTAL randomized trial. *JAMA* 2013, 310:1809-1817.

14. Rackow EC, Falk JL, Fein IA, Siegel JS, Packman MI, Haupt MT: Fluid resuscitation in circulatory shock: a comparison of the cardiorespiratory effects of albumin, hetastarch, and saline solutions in patients with hypovolemic and septic shock. *Crit Care Med* 1983, 11: 839–50.

15. Carli P, Goldstein P, Lejay M, Facon A, Orliaguet G, Petit P: Remplissage vasculaire prehospitalier en taumatologie: hesteril 6% versus plasmion. *Jeur* 2000, 13:101-105.

16. Vlachou E, Gosling P, Moiemen NS: Hydroxyethylstarch supplementation in burn resuscitation--a prospective randomised controlled trial. *Burns* 2010, 36:984-991.

17. Harten J, Crozier JE, McCreath B, Hay A, McMillan DC, McArdle CS, Kinsella J: Effect of intraoperative fluid optimisation on renal function in patients undergoing emergency abdominal surgery: A randomised controlled pilot study. *Int J Surg* 2008, 6:197–204.

18. Ickx BE, Bepperling F, Melot C, Schulman C, Van der Linden PJ: Plasma substitution effects of a new hydroxyethyl starch HES 130/0.4 compared with HES 200/0.5 during and after extended acute normovolaemic haemodilution. *Br J Anaesth* 2003, 91:196–202.

19. Jover JL, García JP, Martínez C, Espí A, Gregori E, Almagro J: [Hydroxyethyl starch to protect renal function in laparoscopic surgery]. *Rev Esp Anestesiol Reanim* 2009, 56:27–30.

20. Fenger-Eriksen C, Hartig Rasmussen C, Kappel Jensen T, Anker-Møller E, Heslop J, Frøkiaer J, Tønnesen E: Renal effects of hypotensive anaesthesia in combination with acute normovolaemic haemodilution with hydroxyethyl starch 130/0.4 or isotonic saline. *Acta Anaesthesiol Scand* 2005, 49:969–74.
