# Supplementary material for: Hydroxyethyl starch versus other fluids for non-septic patients in the intensive care unit: a meta-analysis of randomized controlled trials
Source: Crit Care. 2015 Mar 19;19(1):92. doi: 10.1186/s13054-015-0833-9 (PMC4404666; doi:10.1186/s13054-015-0833-9)
Supplement: Additional file 2: Table S1. — Observation period for outcomes. [file 13054_2015_833_MOESM2_ESM.doc]

**Additional file 2: Table S1: Observation period for outcomes**

| Author, year | Mortality | RRT | Blood loss | RBC transfusion | Fluid application | ICU stay (d) | Hospital stay (d) |
| --- | --- | --- | --- | --- | --- | --- | --- |
| Ley, 1990 [22] | — | — | Hospital | Hospital |  | HES, 2.1±0.3  Crystalloid, 2.8±0.5 | HES, 10.1±1.5  Crystalloid, 10.0±1.9 |
| Beards,1994 [36] | Hospital | — | — | — |  | — | — |
| Van der Linden, 2005 [23] | Postoperatively | — | From surgery to 20h in ICU | NS | The same to blood loss. | HES, 1 (0.8–3.0)  Gelatin, 1.8(0.9–4.2) | HES, 8 (6–24)  Gelatin, 9 (7–35) |
| Chen, 2006 [37] | — | Hospital | — | — | 48h in ICU | — | — |
| Mahmood,2007 [24] | 30d | NS | From 8h before surgery to 24h in ICU | The same to blood loss. | The same to blood loss. | — | — |
| Godet, 2008 [25] | 90d | — | Hospital | — | — | HES, 1 (1–33)  Gelatin, 1 (0–7) | HES, 10 (6–48)  Gelatin, 10 (6–24) |
| Mukhtar, 2009 [26] | Hospital | ICU | — | From surgery to 4d in ICU | From surgery to 4d in ICU | — | — |
| Ooi, 2009 [27] | Hospital | ICU | 24h in ICU | — | 24h in ICU | HES, 2.4±1.4  Gelatin, 2.6±1.3 | HES, 6.1±2.6  Gelatin, 6.0±2.2 |
| Schramko,2009 [28] | — | — | 18h in ICU | 18h in ICU | 18h in ICU | — | — |
| Choi,2010 [29] | — | — | 24h in ICU | NS | 24h in ICU | HES, 1.7±0.8  HA, 1.4±0.6 | HES, 12.0±3.7  HA, 11.6±3.0 |
| Gondos, 2010 [38] | ICU | — | — | — | — | — | — |
| Heradstveit, 2010 [39] | 365d | — | — | — | 24h in hospital | — | — |
| Inal, 2010 [40] | ICU | — | — | — | — | HES, 21.3 ±26.1  Gelatin, 22.1 ± 26.9 | — |
| Schramko,2010 [30] | — | — | 18h in ICU | — | 18h in ICU | — | — |
| Du, 2011 [41] | Hospital | Hospital | — | — | 8d in hospital | — | HES, 25.4 ±16.0  RL, 26.3 ± 16.0 |
| James,2011 [42] | 30d | 30d | — | 24h in ICU | 24h in ICU | — | — |
| Lee, 2011 [31] | — | ICU | 24h in ICU | 24h in ICU | During surgery and 24h in ICU | HES, 2.7±0.8  Crystalloid, 2.7±1.1 | HES, 10.9±5.7  Crystalloid, 10.3±3.7 |
| Yang, 2011 [32] | Hospital | — | — | — | 5d in ICU | HES, 1.2±0.7  RL, 1.3±0.8  HA, 1.2±0.8 | HES, 8.6±1.3  RL, 7.6±0.6  HA, 7.6±0.9 |
| Nagpal, 2012 [34] | Hospital | Hospital | — | — | — | — | — |
| Myburgh, 2012 [6] | 90d | — | — | — | — | — | — |
| Alavi, 2012 [33] | — | — | 24h in ICU | 24h in ICU | 24h in ICU | HES, 45 (42–48)  RL, 46 (42–48)  Gelatin, 47 (43–48) | — |
| Kimenai,2013 [35] | — | — | Before removing drains | — | — | — | — |

Abbreviations and explanation: d, day. “—” means the item unreported in the study. NS, not stated.
